# Autologous Cellular Method Using Micrografts of Human Adipose Tissue Derived Follicle Stem Cells in Androgenic Alopecia

**DOI:** 10.3390/ijms20143446

**Published:** 2019-07-13

**Authors:** Pietro Gentile

**Affiliations:** Surgical Science Department, Plastic and Reconstructive Surgery Unit, University of “Tor Vergata”, 00173 Rome, Italy; pietrogentile2004@libero.it; Tel.: +39-3388515479

**Keywords:** micro-grafts, hair stem cells, hair mesenchymal stem cells, intra dermal-adipose tissue derived follicle stem cells, extra dermal-adipose tissue derived follicle stem cells

## Abstract

Hair bio-engineering has risen at the crossing point of various manipulations to meet a clinical requirement for innovations to advance hair growth. The authors reported the microscopic and trichoscopic results of an autologous cell biological technique to compare, through histological, immunocytochemistry, and cytospin analysis, hair re-growth obtained by micro-grafts from scalp tissue containing Human Intra- and Extra-Dermal Adipose Tissue-Derived Hair Follicle Stem Cells (HD-AFSCs) versus placebo (saline solution). An autologous solution of micro-grafts was obtained from mechanical fragmentation and centrifugation of scalp biopsy’s (2 × 2 mm) using “Gentile protocol”. The micro-grafts solution was mechanically infiltrated on half of the selected patients’ scalps with Androgenic Alopecia (Norwood–Hamilton 2–5 and Ludwig 1–2). The other half was infiltrated with saline solution. Three injections were performed to each patient at 45-day intervals. Of the 35 patients who were enrolled, 1 was excluded and 1 was rejected. 23 and 44 weeks after the last micro graft’s injections, the patients displayed a hair density improvement, with a mean increment of 33% ± 7.5% and 27% ± 3.5% respectively, contrasted with baseline values, for the treated region. Microscopic assessment appeared, in scalp biopsies, to show an expansion in the number of hair follicles per mm^2^ following 11 months from the last micro-grafts application compared with baseline (1.4 + 0.27 versus 0.46 + 0.15, respectively; *p* < 0.05). HD-AFSCs contained in micro-grafts may represent a safe and effective alternative therapy option against hair loss.

## 1. Introduction

For tissue engineering promoting hair re-growth, the use of autologous cell biological technique (A-CBT) based on micro-grafts containing Hair Follicle Mesenchymal Stem Cells (HF-MSCs) obtained by intra- and extra-dermal tissue of the scalp, called, for the first time in this work, “Human Intra and Extra Dermal Adipose Tissue-Derived Hair Follicle Stem Cells (HD-AFSCs)”, has not been adequately considered.

In this study, HD-AFSCs are considered the cellular population containing HF-MSCs and Hair Follicle Epithelial Stem Cells (HF-ESCs).

Androgenic alopecia (AGA) is a dynamic and chronic hair loss, interestingly affecting 80% of white men and 40% of women under age 70 [1,2,3]. Actual medications affirmed for AGA are Minoxidil^®^, Finasteride^®^, and hair transplant [2]. The potential effect of autologous Platelet-Rich Plasma (A-PRP) has been exhibited in later papers [3,4].

In AGA, the miniaturization of the follicles is characterized by a diminishment of anagen and an improvement in resting hair follicles (HFs) telogen phase [5]. Moreover, invading lymphocytes and mast cells were identified around the miniaturizing follicle [6] and in the lump zone, rich with stem cells (SCs) [7]. In a hair loss scalp, HFs-SCs numbers stay unaltered, though the number of more actively proliferating progenitor cells particularly diminish [8]. This proposes that a bald scalp either does not have an activator or has an inhibitor of HFs growth.

Two years ago (2017), a previous work [9] detailed the preliminary utilization of small autologous micro-grafts, obtained through the disaggregation of punch’s biopsy of the scalp, to increase the hair’s thickness in eleven patients influenced by AGA. Specifically, in the previous work, the main mechanical detachment of human follicle SCs not extended and obtained by a slow centrifugation as per the negligible manipulation rules was accounted for. Other studies have established a role of HF-MSCs and autologous micro-grafts in the improvement of hair density in patients affected by AGA [10,11,12].

Right now, in this examination, we researched as A-CBT the in vivo execution of micro-grafts containing HF-MSCs in hair-regrowth, detailing the number of HFs per mm^2^ following 11 months from the treatment contrasted with baseline and the long-haul clinical assessment.

HD-AFSCs can be isolated by enzymatic digestion or mechanical disaggregation. In the first technique, the scalp fragment is reaped sterilely, processed on proper catalysts, and after that, the resulting cell solutions are seeded in culture dishes containing a unique medium supplemented with vital added substances, and afterward incubated. At long last, the subsequent colonies are sub-cultured before conversion and the cells are fortified to differentiate [9,10,13]. According to the European rules (EC n. 1394/2007) and European Medicine Agency (EMA)/Committee for Advanced Treatments (CAT) recommendations (EMA/CAT/600280/2010 Rev 1), it is possible to perform a substantial manipulation of cells expansion in culture and re-injection of the expanded cellular suspension obtained only through Good Manufacturing Practices (GMP) laboratories with Clinical Trial authorized by a Health Ministry. In fact, the suspension of cells expanded after substantial manipulation is considered an advanced therapy medicinal product. On the other hand, a cellular suspension obtained by enzymatic digestion without cells expansion in culture (only cells isolation), or by mechanical fragmentation, are not considered substantial manipulation in that biological characteristics, physiological functions, or structural properties relevant for the intended clinical use are not altered and in addition they must contain cells that are intended to be used for the same essential function(s) in the recipient area and the donor area. The EMA/CAT considers that the products obtained through this modality of preparation does not fall within the definition of an advanced therapy medicinal product, as provided in Article 2 of Regulation (EC) 1394/2007. In addition, enzymatic digestion procedures have longer preparation times and higher costs. For these reasons, the mechanical fragmentation procedure was preferred to cell expansion or enzymatic digestion procedures.

In the second technique, autologous micro-grafts containing HD-AFSCs can be isolated by the “Gentile procedure” in which the scalp is dealt with as some other connective tissue subjected to grafts, with a period of collection and a period of mechanical disaggregation of the tissue without controlling the grid. The “Gentile procedure” can be performed using a medical device, as previously reported [9] or without, through a procedure of scalp tissues’ splitting producing a great many practical micro-grafts and filters them with a cut-off size of 80–140 microns, to advance the releasing of old separated cells and the improvement of youthful ancestor cells contained inside the scalp tissue [9,10,13,14].

HF-MSCs exhibited surface markers of bone marrow mesenchymal stem cells, as shown by positive staining for CD44, CD73, CD90, and CD105, and they also displayed trilineage differentiation potentials into adipocytes, chondrocytes, and osteoblasts by cytochemistry and qRT-PCR [10].

The human HFs bulge is also an important niche for keratinocyte stem cells and epithelial stem cells (ESCs) [13]. Positive markers for bulge cells included CD200, follistatin, PHLDA1, and frizzled homolog 1, while CD34, CD24, CD71, and CD146 were preferentially expressed by non-bulge keratinocytes, as reported by Ohyama et al. [13]. In addition, CD200^+^ cells (CD200hiCD24loCD34loCD71loCD146lo) obtained from hair follicle suspensions demonstrated high colony-forming efficiency in clonogenic assays, indicating successful enrichment of living human bulge stem cells [13]. For this reason, the authors analyzed for the expression of mesenchymal/epithelial stem cells markers, CD44 [10] and CD200 [13] respectively, for the recognizable proof of HD-AFSCs.

This study intends to clear up the clinical and microscopic impacts of micro-grafts scalp infusion in people with AGA improving the use of A-CBT. It could enhance hair re-growth, subsequently making the micro-grafts technique a contrasting option to Finasteride^®^, Minoxidil^®^, or different medications and creams. The information we have reported exhibits the safety and efficacy of the procedure.

## 2. Results

### 2.1. Long-Haul Clinical Results

**Figure 1:**
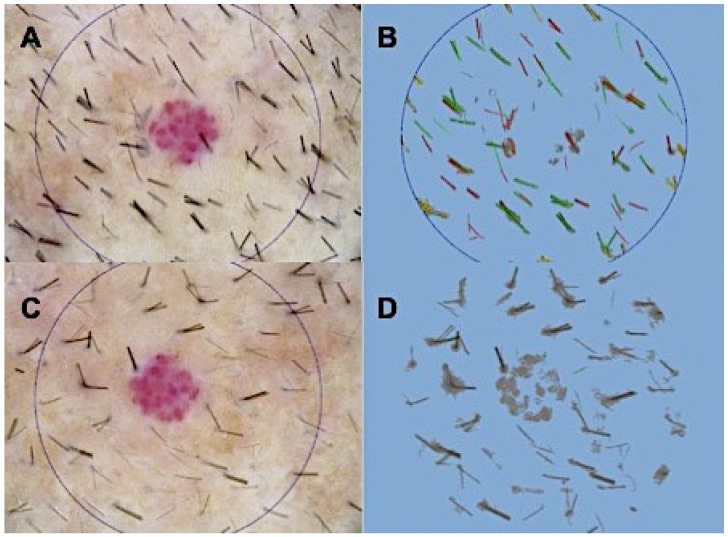
Trichoscan digital image analysis performed by Fotofinder in a patient shown in Figure 2. (**A**,**B**) At T5 post-operative hair count was 87.5 hairs/0.65 cm^2^, and proportions of telogen and anagen hairs were 50.6% and 47.5%, respectively. (**C**,**D**) At T0 pre-operative hair count was 69.2 hairs/0.65 cm^2^, and proportions of telogen and anagen hairs were 39.4% and 57.0%, respectively.

At T4, 23 weeks after the last treatment with micro-grafts containing HD-AFSCs, the mean hair density increments were 33% ± 7.5% over baseline values for the treated region and less than 1% increment for the region infused with saline. At T0 (Figure 1C,D), no statistical contrasts in hair density or hair thickness existed between the treated region and placebo area. At T5, 44 weeks after the last micro-grafts injection, the mean hair density increments reported by phototrichograms were 27% ± 3.5% (Figure 1A,B) over baseline values for the treated region and not more than a 0.7% increment in hair density for the placebo region.

Relapse of hair loss was not assessed in treated patients until a year after the last injection. In the following 16 months, 3 patients detailed dynamic hair loss. Those three patients were re-treated.

**Figure 2:**
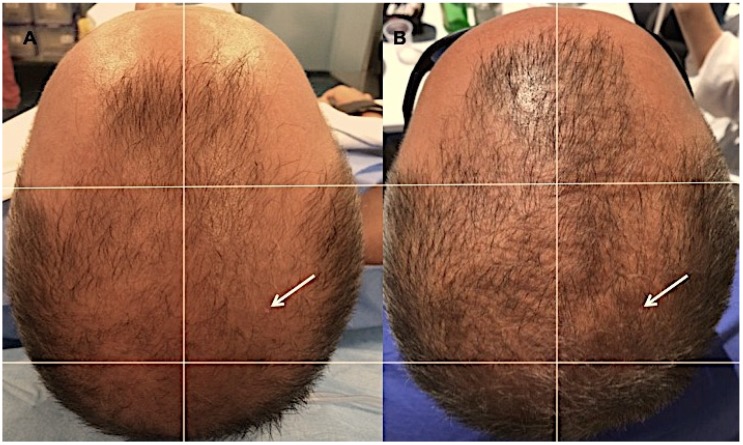
A smoker 43-year-old male patient classified as Androgenic alopecia (AGA) 4 according to the Norwood–Hamilton Scale, in baseline and after Human Intra and Extra Dermal Adipose Tissue-Derived Hair Follicle Stem Cells (HD-AFSCs) treatment. (**A**) Timing T0 (before the treatment) with hair loss localized to the vertex, parietal, temporal, and frontal areas, (**B**) Timing at T5, 44 weeks after the last micro-grafts injection with an increase of hair density in the targeted area. White arrow indicates the demarcated point in which the histological analysis and Trichoscan evaluation were performed.

**Figure 3:**
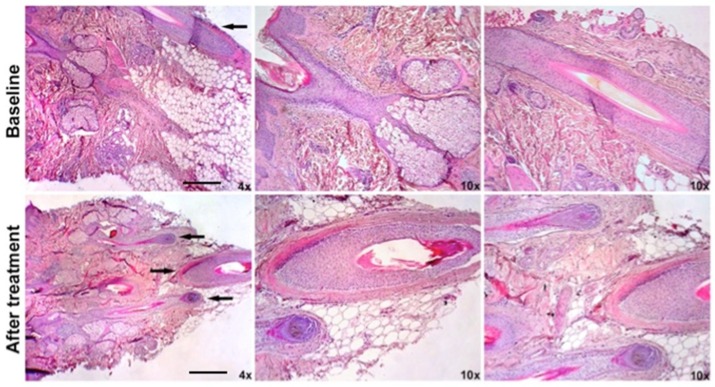
Microscopic evaluation of pattern hair loss after micro-grafts application. Representative microphotographs of scalp biopsies at baseline (time 0) and at 44 weeks after the micro-grafts treatment. Arrows show hair follicles. Magnification 4× and 10×.

In this examination, we demonstrated the long-haul clinical impact of the infusion of micro-grafts suspension, as shown in Figure 1A–D and Figure 2A,B reporting an improvement in the formation of new follicles (Figure 3).

### 2.2. Immunophenotypic Identification and Cell Counting

**Figure 4:**
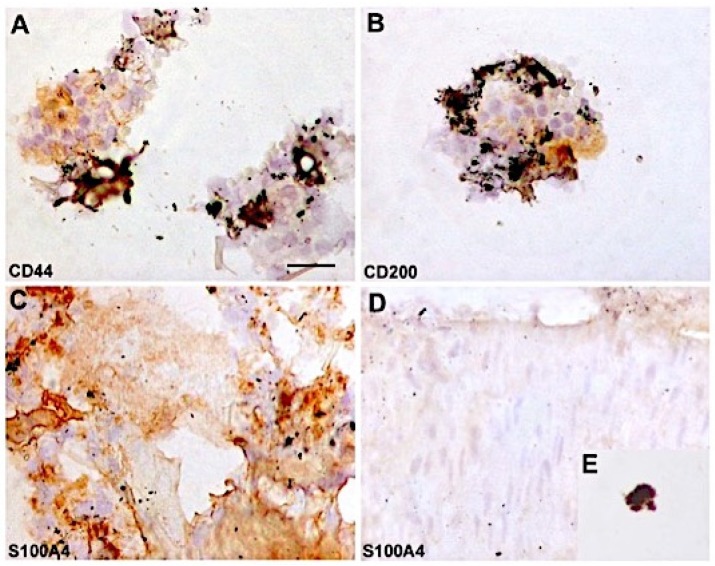
Immunophenotypic characterization of micro-graft’s suspension containing HD-AFSCs. (**A**,**B**) Immunocytochemistry for mesenchymal and epithelial stem cells markers, CD44 and CD200, respectively. (**C**) The positive expression of S100A4 in dermal fibroblasts. (**D**) The immunonegativity of epithelial cells. (**E**) Some melanocytes are shown in detail. Scale Bars 50 μm. Magnification 40×.

Each 1.1mL of micro-grafts solution, contained a mean of about 5,233.9 + 521.9 cells. In particular, the percentage of HF-MSCs CD44^+^ (from dermal papilla) was about 6.1% + 0.2% whereas the percentage of HF-ESCs CD200^+^ (from the bulge) was 2.9% + 0.8%. Thus, the aggregate percentage of HD-AFSCs in the micro-grafts suspension was around 9.0% ± 1%. The rest of the cells were, for the most part, S100A4+ dermic fibroblasts (>84%) and epidermal cells (melanocytes and epithelial cells <10%), conspicuous by their trademark morphological aspects (Figure 4A–E). No positivity was recognized for CD31 expression.

### 2.3. Histological and Microscopic Analyses of Micro-Grafts Procedure

Microscopical assessment (Figure 3) exhibited, in scalp biopsies, an expansion in the number of HFs per mm^2^ following 44 weeks from the micro-grafts treatment contrasted with baseline (1.4 ± 0.27 versus 0.46 ± 0.15, individually; *p* < 0.05).

### 2.4. CD34, CD200 and KRT15 Expression Levels in Flow Cytometry, Comparing Scalp Treated with HD-AFSCs Versus Baseline

CD200, ITGA6, and CD34 cells were greatly improved after HD-AFSCs treatment. These cells likely represent early progeny of stem cells, based on their position in the follicle, stem cell marker expression levels, cell size, and cell cycle state. These results suggest that improvement of progenitor cells contributes to human hair re-growth.

**Figure 5:**
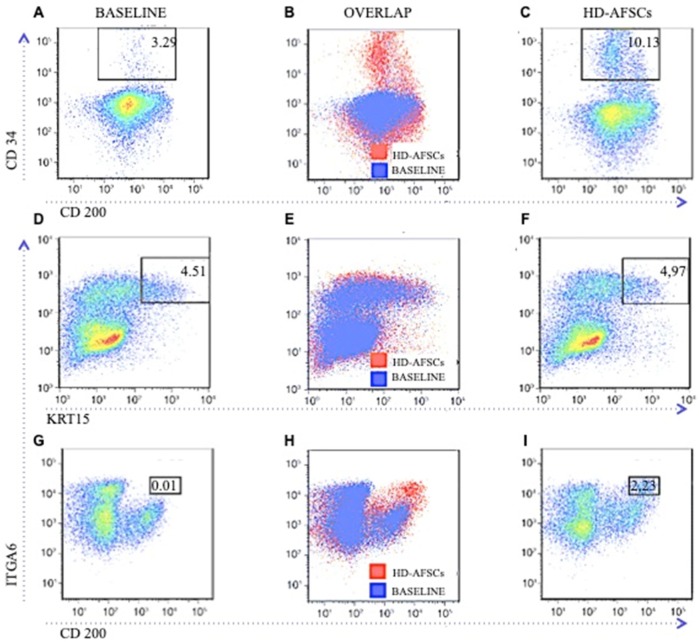
Preservation of KRT15 hair follicle stem cells in baseline and after HD-AFSCs treatment, with improvement of CD200, ITGA6, and CD34 progenitor cells in targeted area. Pseudocolor dot plots (**A**,**C**,**D**,**F**,**G**,**I**) or overlap graphs (**B**,**E**,**H**) from FACS analysis of epithelial cells stained with the indicated antibodies. Overlap graphs are plotted first with cells from HD-AFSCs-treated scalp, then layered over with cells from baseline. (**D**–**F**) Representative example showing KRT15 cells preserved in baseline (*p* = 0.5). In contrast, a defined population of CD200 ITGA6 cells (**G**–**I**; *p* = 0.003) and CD34 cells (**A**–**C**; *p* = 0.011) were improved in HD-AFSCs-treated area. Numerical values represent percent cells in the gated population, of all cells plotted in the representative experiments.

Knowing that cells with the highest level of KRT15 expression possess epithelial stem cell properties [15], it was next addressed whether hair follicle stem cell numbers improve in the targeted area of the scalp after HD-AFSCs versus baseline, in this study. Single cell-suspensions of epithelial cells from the scalp at baseline and after HD-AFSCs treatment from the same patients were isolated and stained with antibodies against ITGA6 and KRT15 to be analyzed by flow cytometry (Figure 5). In each paired sample from the same individual, an identical gate defining the top 5% of cells in the treated scalp was applied to the cells from the scalp at baseline. On average, the percentage of KRT15 cells was the same in the scalp at baseline and after HD-AFSCs treatment (Figure 5D–F; 4.5% ± 0.6% versus 4.9% ± 0.07%, *p* = 0.5). To define changes in CD200^+^ cells in men with AGA, we analyzed CD200 expression together with expression of the epithelial basal cell marker ITGA6 by flow cytometry in the matched baseline and treated scalp. It was found that a well-demarcated population of cells expressing high levels of both CD200 and ITGA6 was markedly improved in the targeted area treated with HD-AFSCs versus baseline (Figure 5G–I; 2.2% ± 0.5% versus 0.01% ± 0.1%, *p* = 0.003,). This population represented 10.0% ± 0.1% of the entire CD200^+^ population. To better characterize CD200 and ITGA6 cells with respect to their stem cell properties, we determined their level of KRT15 expression, cell size, and degree of quiescence. We compared cells gated as CD200 and ITGA6 (Figure 5I), as well as cells gated as KRT15 ITGA6 (Figure 5F), with an otherwise ungated population of all ITGA6 cells. To assess whether other progenitor cell populations were improved in treated scalps, we quantitated the number of CD34+ cells, which juxtapose the bulge and localize below it in the outer root sheath. We found that CD34+ cells were improved roughly 3-fold in targeted area versus baseline (Figure 5A–C; 3.2% ± 1% versus 10.1% ± 0.3%, *p* = 0.011).

## 3. Discussion

Right now, AGA includes a yearly worldwide market income of 4 billion dollars and a growth rate of 1.8%, demonstrating a developing consumer market [16]. The reconstitution of a completely sorted out and utilitarian HFs from separated cells propagated under characterized tissue culture conditions is a test as yet pending in hair-tissue engineering [17].

Therefore, it is of extraordinary enthusiasm to discover distinctive methods planning to neo-generate the HFs with appropriate conditions of a grown-up person. In light of the actual knowledge on the dermal and epithelial cells and their associations amid the embryonic hair generation and grown-up hair cycling (HC), numerous scientists have endeavored to acquire mature HFs utilizing distinctive methods and methodologies relying upon the reasons for AGA [17].

In the preparatory investigation [9], another strategy was built up based on A-CBT, to disengage human grown-up SCs by disaggregation of punch biopsy from intradermal adipose tissue without culture and/or enzymatic digestion, reporting the cell counting and the preparatory outcomes, acquired by the micro-grafts infusions in targeted areas presenting AGA, enhancing hair thickness.

Specifically, in this past work, the percentage of CD44^+^ HF-MSCs, from dermal papilla, and the percentage of CD200^+^ HF-ESCs, from the lump, obtained via programmed centrifugation of 11 punch tests [9] using Rigenera^®^ CE medical device (CE certified Class I, product by HBW Srl; Turin, Italy) was detailed. Starting with this experience, the authors now feel the necessity to compare the results obtained by the “Gentile procedure” which performed a mechanical fragmentation and disaggregation of scalp’s tissue through a splitting and centrifugation phases versus the preliminary results obtained by the Rigenera^®^ procedure, which only performed a centrifugation of scalp’s tissue. As reported in the results section, with the “Gentile procedure” (three injections each 45 days), at T4, 23 weeks after the last treatment with micro-grafts containing HD-AFSCs, the mean hair density increments were 33% ± 7.5% over baseline values for the treated region, while at T5, 44 weeks after the last micro-grafts injection, the mean hair density increments reported by phototrichograms were 27% ± 3.5% over baseline values for the treated region. Each 1.1mL of micro-grafts solution, contained a mean of about 5,233.9 ± 521.9 cells, where the percentage of HF-MSCs CD44^+^ (from dermal papilla) was about 6.1% ± 0.2% while the percentage of HF-ESCs CD200^+^ (from the bulge) was 2.9% ± 0.8%. Thus, the aggregate percentage of HD-AFSCs, in the micro-grafts suspension, was around 9.0% ± 1%. In the previous work [9] based on the use of the Rigenera^®^ procedure, 1.2 mL of micro-grafts solution, contained a mean of about 3,728.5 ± 664.5 cells, where the percentage of HF-MSCs CD44^+^ was about 5.0% ± 0.7% while the percentage of HF-ESCs CD200^+^ was 2.6% ± 0.3% [9]. Regarding the clinical outcomes obtained by the Rigenera^®^ procedure and reported in the previous work [9], 23 weeks after the last treatment with micro-grafts (two injections each 60 days), the mean hair density increments were 29% ± 5% over baseline values for the treated region.

In addition, it was revealed in this work that the microscopic assessment performed by cytospin, immunocytochemistry, histological examination obtained by Haematoxylin-Eosin-stained and long-haul clinical assessment, should be talked about as follows: current improvement in the distinctive methodologies to neo-generate HFs, with an accentuation on those including neo-genesis of HFs in grown-up people utilizing isolated cells and biotechnologies.

Investigations were performed utilizing rat cells, especially from embryonic or infant origin [17]. Notwithstanding, no successful methodology to neo-generate human HFs from grown-up cells has yet been accounted for. Maybe the most critical challenge is to give three-dimensional culture conditions mirroring the structure of living tissue. Enhancing culture conditions that permit the extension of particular cells while securing their inductive properties, and additionally, the strategies for choosing populaces of ESCs should give us the fundamental tools to overcome the challenges that constrain human HFs neo-genesis [17].

These cells give off an impression of being situated in the lump region of HFs.

HFs are known to contain a well-characterized niche for grown-up SCs: the lump, which contains ESCs and melanocytic SCs [18]. SCs in the hair lump, an obviously differentiated structure inside the lower permanent portion of HFs, can generate the inter-follicular epidermis, HFs structures, and sebaceous glands [19,20]. The lump ESCs can likewise reconstitute in a simulated in vivo framework to a new HF [21,22]. Yu et al. [18] demonstrated that human HFs contain a SCs populace that can be separated into the smooth muscle cell, neuron, and melanocyte heredities in induction medium. Their information demonstrates that Oct4-positive cells are available in human skin, and the majority of them are situated in the HFs in vivo [18,23]. Subsequently, human HFs contain multipotent SCs other than ESCs and melanocytic SCs, and these cells are situated in the lump region. These cells indicate promising plasticity in ex vivo and in vitro conditions, making them potential candidates for cell-engineering and cell-substitution treatments. Each mature HF is a regenerating framework, which physiologically experiences cycles of growth (anagen), relapse (catagen), and rest (telogen) at various times in a grown-up’s life [24]. In catagen, HFs-SCs are kept up in the lump. At that point, the resting follicle re-enters anagen (regeneration) when legitimate molecular signals are given. Amid late telogen to early anagen change, signals from the Dermal Papilla (DP) stimulate the hair germ and quiescent lump stem cells to wind up activated [25]. Numerous paracrine factors are engaged with this crosstalk at various HC stages and some signaling pathways have been implicated [26,27,28]. In anagen, SCs in the lump offer ascent to hair germs, at that point the transient increasing cells in the grid of the new follicle proliferate quickly to frame another hair filament [29]. As a matter of fact, the authors feel the need to better understand in which stage it is critical to act. Regeneration of HFs was likewise seen in people [30] when dermal sheath tissue was utilized, which was adequate to additionally regenerate the DP structure. After implantation, the whisker DP was equipped for promoting HFs regeneration holding the data to decide hair fiber type and follicle size [31]. Grafting of dermal-inductive tissue was restricted by the way that it was impractical to produce more HFs than the one obtained from the donor tissues. To defeat this constraint, diverse methodologies and exploratory models utilizing freshly or cultured isolated cells from both dermal and dermal/epidermal origin were tried [31]. The vast majority of them included neonatal and embryonic murine cells [31]. In an examination published in 2015 by Balañá ME et al. [17] the writers prepared a dermal-epidermal skin substitute in a research facility by seeding an a-cellular dermal grid with cultured HFs-ESCs and dermal papilla cells (DPCs), both obtained from the grown-up human scalp. These constructs were grafted into a full-thickness wound produced on bare mice skin. In fourteen days, histological structures reminiscent of a wide range of phases of embryonic HFs improvement were seen in the grafted region. These structures demonstrated concentric cellular layers of human origin and expressed k6hf, a keratin present in epithelial cells of the companion layer. Despite the fact that the presence of completely mature HFs was not observed, these outcomes demonstrated that both epithelial and dermal cultured cells from the grown-up human scalp in a dermal scaffold could create in vivo structures that reiterate embryonic hair improvement. In a current report published in 2017 by Kalabusheva et al. [32], postnatal human dermal papilla (DP) cells and skin epidermal keratinocytes (KCs) in a hanging drop culture to build up a simulated HFs germ were combined. The technique depends on DP cell hair-inciting properties and KC self-association. They assessed two protocols of total collecting. Blended HF germ-like structures showed the initiation of epithelial–mesenchymal collaboration, including WNT pathway enactment and expression of follicular markers [32]. They examined the impact of conceivable DP cell niche components including dissolvable components and extracellular matrix (ECM) molecules during the time spent on the organoid assembling and growth. Their outcomes showed that soluble components had little effect on HF germ generation and Ki67+ cell score inside the organoids despite the fact that BMP6 and VD3 effectively kept up the DP character in the monolayer culture. Aggrecan, biglycan, fibronectin, and hyaluronic acid (HA) significantly stimulated cell proliferation in DP cell monolayer culture with no impact on DP cell character [32]. A large portion of ECM compounds restricted the growth of cell totals while HA advanced the formation of bigger organoids [32]. Talavera-Adame et al. [16], revealed in a recent study the biomolecular pathway involved in a cellular treatment. Specifically, it has additionally been demonstrated that Wnt/β-catenin signaling is fundamental for the growth and upkeep of DPCs [33,34]. The increment of Wnt signaling in DPCs evidently is one of the principal factors that enhanced hair growth [33]. Another fascinating field in A-CBT, is the likelihood to utilize the fat graft and adipose-derived stromal vascular fraction cells (AD-SVFs) in hair re-growth. The autologous fat grafting method is >100 years old, with a current and sensational increment in clinical experience in the course of the last 10–15 years [35]. AD-SVFs is a heterogeneous group of non-cultured cells that can be dependably removed from fat of the body (not intra-dermal adipose tissue of the scalp) by utilizing computerized frameworks, in light of centrifugation, filtration, and purification of fat tissue or utilizing enzymatic absorption (not proposed by EMA-CAT recommendations). These cells work to a great extent by paracrine systems to help adipocyte viability. Considering that the quality and quantity of adipose-derived stem cells strictly depends on the adipose tissue or lipoaspirate handling techniques, patients’ variability, purifying method, and storage condition [36,37] appears important to identify the suitable procedure (for the country regulations, and for the A-CBT available) analyzing the most recent experiences in term of results obtained by different authors. Festa et al. detailed in 2011 that adipocyte ancestry cells bolster the SCs niche and help drive the complex hair growth cycle [35]. This follicular regenerative approach is fascinating and raises the likelihood that one can drive or reestablish the HC in male and female hair loss by stimulating the niche with autologous fat improved with AD-SVF [35]. Along these lines, Perez-Meza D et al. [38] detailed the safety, tolerability, and quantitative, in a study group of 9 people with early hereditary alopecia treated with subcutaneous scalp infusion of advanced fat tissue. In this pilot case arrangement, a mean increment of 31 hairs/cm^2^ of the scalp (represents a 23% relative percentage increment) was recorded in patients experiencing treatment of fat blended with AD-SVF. In the examination, one subject who had fat alone reported an increment of 14 hairs/cm^2^ of the scalp, proposing that while fat alone may represent an approach for early hairlessness, the addition of AD-SVF may improve this reaction [38]. The discoveries propose that scalp stem cell-enriched fat grafting may represent a promising elective way to deal with treating hair loss in people. Fukuoka et al. [39] inspected the impacts of fat-derived SCs-conditioned medium infusion in twenty-two patients (eleven men and eleven women) with alopecia. Patients got treatment each 3 to 5 weeks for a total of 6 sessions. The mean increment in the hair count was 29.0 ± 4.1 in men and 15.6 ± 4.2 in women. No noteworthy distinction was seen amongst men and women.

The examination of all these investigations could prompt the conclusion that HFs neo-genesis utilizing human epithelial and dermal cells is an extremely cumbersome assignment that could require unique culture conditions, some way or another reproducing the ordinary or embryonic skin condition, and the utilization of embryonic or neonatal cells.

## 4. Methods

This retrospective observational case-series, randomized, evaluator-blinded, placebo-controlled, half-head group study was performed following the principles outlined in the Declaration of Helsinki and internationally consented ethics in clinical research [40]. A quality assessment was carried out based on the STROBE checklist [41] and CONSORT (Consolidated Standards of Reporting Trials) flow diagram [42].

The protocol was conducted in strict adherence to the European Rules and institutional guidelines represented by EMA/CAT/600280/2010 Rev 1, Committee for Advanced Treatments (CAT) and EC n. 1394/2007.

The study protocol, object of a research contract (D.R. 1467/2017) and a university masters degree called “Regenerative surgery and medicine in wound care management”, was approved with Rectoral Degree (D.R. n. 1794/2018) of 19 September 2018 and the Ethics on Research Committee of the School of Medicine, “Tor Vergata” University, Rome, Italy, with registration number #0031036/2018.

All patients received detailed oral and written information about the study, including the risks, benefits, and alternative therapies, and signed an informed consent form before any study procedures.

### 4.1. Patients

**Scheme 1:**
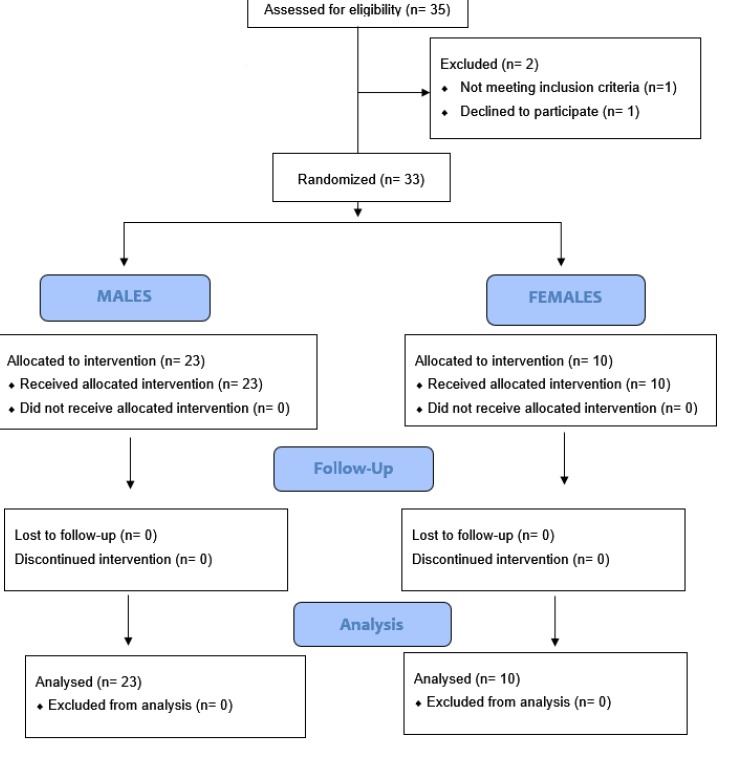
CONSORT (Consolidated Standards of Reporting Trials) flow diagram.

This investigation enlisted 35 patients, age 21–70 years, 23 males who showed AGA of grade 2–5 as controlled by the Norwood–Hamilton classification scale and 12 females affected by AGA with grade 1–2 as dictated by the Ludwig classification scale. Of the 35 patients enrolled, 1 was excluded and 1 was rejected. Quality assessment was reported in a CONSORT flow diagram (Scheme 1). Systemic and local exclusion criteria were considered. Fundamental and local prohibition criteria were considered. Fundamental expulsion criteria included immunosuppression and cancer, sepsis, and also the utilization of pharmacological therapeutics targeting AGA (Finasteride^®^, similar drugs, and/or anti androgens) in the earlier year. Localized expulsion criteria included the utilization of topical medicines and lotions for androgenic alopecia in the earlier year.

Diagnoses of AGA were established performing:

- Detailed therapeutic history;
- Clinical examination (increment in miniaturization or diminished hair number, alongside negative hair pull tests);
- Blood test and urinalysis (1. to expel elective reasons for hair loss, for example, poor nourishment, iron deficiency, thyroid dysfunction, and syphilis; 2. to distinguish percentages of 17-idrocorticosteroid, 17-ketosteroid, dehydroepiandrosterone, free cortisol, pregnanetriol, testosterone, cortisol, dihydrotestosterone, DHEA, D4-androstenedione, 17-hydroxyprogesterone, 3-α-diol glucuronide, prolactin, and gonadotropins);
- Trichoscopic highlights (>20% fluctuation in hair measurement amongst affected and unaffected zones).

### 4.2. Preparation of Autologous Micro-Grafts Suspension

**Figure 6:**
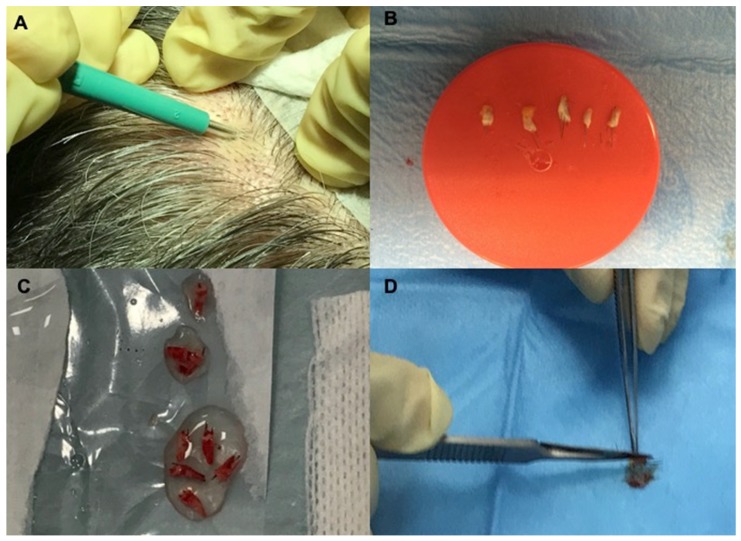
Micro-grafts preparation. Step 1 (Punch biopsy harvesting and fragments selection) (**A**) punch biopsy (2 mm of diameter), (**B**) fragments selections containing intact bulb and bulge area, (**C**) fragments stored in saline solution, (**D**) splitting of the scalp tissues harvested into the strips (2.0 × 2.0 mm).

**Figure 7:**
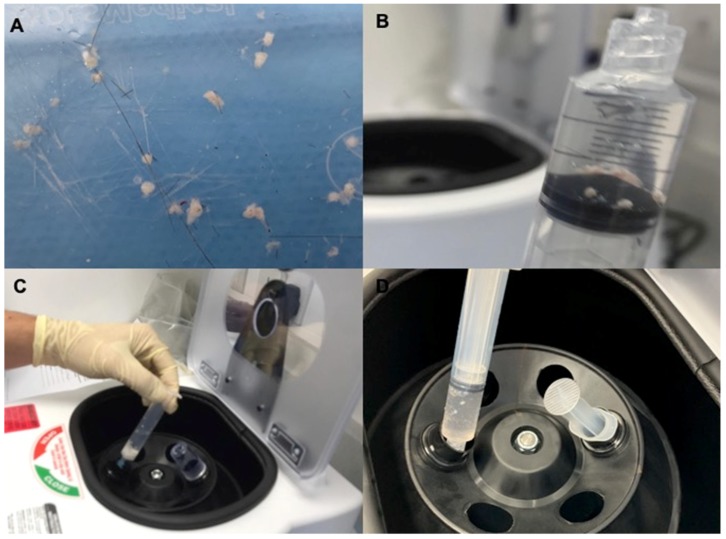
Micro-grafts preparation. Step 2 (Scalp tissues centrifugation). (**A**) Fragments split, (**B**) harvesting of split fragments stored in saline solution, (**C**) centrifugation of two 10mL luer-look syringes containing split fragments, (**D**) solution obtained.

**Figure 8:**
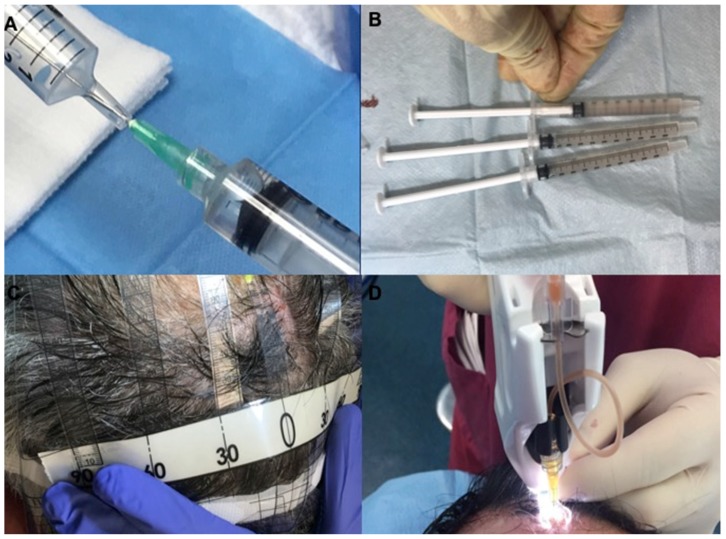
Micro-grafts preparation. Step 3 (Collection of micro-grafts’ suspension and infiltration). (**A**) Removing 1.5 mL of supernatant, (**B**) detail of syringes containing micro-grafts suspension, (**C**) the selected area of the scalp treated identified by specific centimeter, (**D**) mechanical and controlled infiltration performed by mesotherapy gun.

Autologous micro-grafts of HD-AFSCs for clinical use were prepared using the “Gentile procedure” (Figure 6, Figure 7 and Figure 8). Briefly, this procedure represents an innovative clinical approach to obtain autologous micro-grafts through a mechanical fragmentation of different biological tissues [14] and requires different steps of execution. First step: harvesting of the scalp tissues (30–50 fragments depending on the size of the area to treat) with punch biopsy (2 mm diameter) (Figure 6A), selecting the fragments presenting an intact bulb and bulge area (Figure 6B) stored in saline solution (Figure 6C) and cutting the fragments into strips of 2.0 × 2.0 millimeters (mm); collecting and disaggregation of the strips (group of 3 fragments each time) sterilely through a manual splitting performed by multiple incisions with scalpel number 11 (Figure 6D) in 1.2 mL of saline (NaCl 0,9% (mE/mL: Na^+^ 0,154; Cl^−^ 0,154); mOsm/L 308, pH 4.5–7.0) for each 3 fragments (Figure 7A) with the aim to sort a cell group having a diameter of 80–140 μm. Second step: collecting the suspension obtained, average 20 mL for 50 fragments disaggregated and fragmented (Figure 7B), in two 10 mL luer-look syringes and centrifugation for 3 min at 3000 RPM (Figure 7C); 1.5 mL of the supernatant was removed for each syringe (Figure 8A) and 9 mL of micro-graft’s suspension was obtained (two 10 mL luer-look syringes each-one containing 4.5 mL of suspension) (Figure 7D); three syringes each one containing 1.1 mL of micro-graft’s suspension (Figure 8B) were collected each time and (for a total of 3.3 mL of micro-graft’s suspension –1.1 mL of suspension was undergone to histological analysis) were positioned in a mesotherapy gun, individually. Third step: identification of targeted area (Figure 8C) and mechanical and controlled infiltration, using 1 ml syringes into the selected area of the scalp through a medical device, mesotherapy gun (Figure 8D), equipped with a software that allows to schedule the depth of injection, the amount of infiltration per cm^2^, with the same angle of inclination.

Samples of micrograft’s solution (1.1 mL of suspension) were cultured and characterized through cytospin and immunocytochemistry to isolate and study the HD-AFSCs. As previously reported, the authors considered HD-AFSCs the cellular population containing HF-MSCs and HF-ESCs.

### 4.3. Protocols and Mechanical Injection of Autologous Micro-Grafts’ Suspension

The scalp was separated into six areas (front right and left, right and left parietal, right and left vertex) as detailed in Figure 5A,B. Anesthesia (local or potentially fundamental) was not performed. The micro-grafts suspension inter-follicular infusions (0.20 mL cm^2^) were realized to targeted areas at a depth of 5.0 millimeters utilizing a mesotherapy gun outfitted with a 1 mL syringe luer-look with needle 30-gauge (Figure 8D), in three sessions spaced 45 days apart.

Micro-grafts suspension infusions were conveyed to the front area of the scalp while saline solution (placebo) was infused in the parietal areas in patients affected by AGA confined to the frontal and parietal areas. In a like manner, for AGA restricted to the vertex and parietal areas, micro-grafts suspension was infused in the parietal area while saline solution was infused in the vertex area. Specifically, to better show the clinical impacts, we partitioned every area on two sides and we performed the infusions in a chosen side (left or right). Equivalent quantities of autologous micro-grafts suspension and placebo infusions were made.

### 4.4. Evaluation of Hair Growth

Assessment of hair growth was assessed in four phases: T0, before the first infusion, T1, in 3 weeks (wks), T2, in 9 wks, T3, in 16 wks, T4, in 23 wks, T5, 44 wks after the last injection (Supplementary Table S1). The hair growth was assessed at baseline and later the last injection (third infusion was performed following 45 days) through photography (same contrast, brightness, distance, and focus) comparing the placebo area with the treated area. The impacts of micro-grafts suspension and saline medications on hair growth were evaluated through phototrichograms and biopsies and, in addition, by the assistance of worldwide photography, doctor’s and patient’s worldwide evaluation scale.

Phototrichograms were performed in all targeted areas using video-epiluminescence microscopy performed by Fotofinder^®^ (Fotofinder^®^ system, http://www.fotofinder.de) combined with the Trichoscan^®^ digital image analysis (Tricholog GmbH and Datinf GmbH, http://trichoscan.com). TrichoScan^®^ was performed to evaluate hair density (hairs/cm^2^), hair count (hairs/0.65 cm^2^), hair thickness, anagen-to-telogen ratio, and vellus hair-to-terminal hair ratio. During TrichoScan^®^ evaluation, all hairs with thickness > 40.0 µm are described as terminal hairs, while hairs with lesser thickness are described as vellus hairs.

In all people treated, 2 translational regions of hair loss, one at the fringe of the treatment half and a moment along the outskirt of the placebo half, were demarcated with a semi-permanent tattoo.

In detail, the phototrichograms for hair assessments and biopsies for histological analysis were performed before and after the procedure, in the same point demarcated with a semi-permanent tattoo or alternatively, in a point demarcated by nevus/angioma or spider nevi, as reported in Figure 1A,C.

### 4.5. Immunocytochemistry and Cytospin

Micro-grafts suspension tests obtained from human scalp tissues (*n* = 33) were processed. Quickly, the samples were fixed with 4% paraformaldehyde, and analyzed for the expression of S100A4 (dermal fibroblasts) and mesenchymal/epithelial stem cells markers, CD44 [10] and CD200 [13] respectively, for the recognizable proof of HD-AFSCs.

After cell bond on a glass slide by cytospin, immunocytochemistry was performed with particular essential antibodies (S100A4 Dako, 1:30; CD44 sc-9960, 1:10; CD200 ab203887, 1:100) [9]. Positive cells were checked in the aggregate region under a light microscope at 400× magnification (Eclipse E600, Nikon, Tokyo, Japan) and representative microphotographs were captured by the DXM1200F Digital camera (Nikon) utilizing ACT-1 programming (Nikon).

### 4.6. Flow Cytometry

Samples analyzed were treated with 5 U/ml Dispase (Sigma-Aldrich, Saint Louis, MO, USA) in agreement with the protocol previously published [15]. Samples were stained with different combinations of antibodies to ITGA6 (BD Biosciences — Pharmingen clone GoH3) and CD200 (MRC OX-104), fixed and permeabilized (Caltag labs), and stained with the anti-human antibodies against actin (Sigma-Aldrich clone AC-15), KRT15, FST, Ki67 (BD Biosciences - Pharmingen clone B56), CD117 (BD Biosciences clone 104D2), CD45 (BD Biosciences clone 2D1), Ber-EP4, DAPI, and/or an isotype control (IgG2a, Sigma-Aldrich). Analyses were realized on a dual-laser flow cytometer (BD FACSCalibur). All flow cytometry data were analyzed by uploading files into FlowJo 4.6 (TreeStar Inc., Ashland, OR, USA) [15]. KRT15, CD200 and CD34 expression were analyzed to assess the stem and progenitor cell compartments in the targeted area treated with HD-AFSCs at baseline and after treatment.

### 4.7. Histological Analyses

Scalp tissue fragments obtained by punch biopsies (2.0 × 2.0 millimeters), taken from 33 patients at baseline (time 0) and following 11 months from the last micro-grafts injection, were fixed in buffered formalin. The morphometric investigation was performed on Haematoxylin-Eosin-stained paraffin serial areas by assessing the number of follicles per mm^2^, as already depicted [4].

### 4.8. Statistical Analysis

Values as the mean in addition to baseline mistake were examined by means of Student’s *t*-test, and contrasts considered statistically noteworthy were *p* < 0.05. A comparison between hair count values (baseline versus T5) and hair density values (baseline versus T5) for each patient was done with Odds Ratio, CI 95%, and Student’s t-test using MEDCALC^®^ statistical software.

## 5. Conclusions

Our information obviously highlights the constructive effects of A-CBT based on micro-grafts infusions on AGA without major side effects. Compared with previous work published in 2017, the “Gentile procedure” showed better results in term of clinical and histological outcomes. Micro-grafts containing HD-AFSCs may serve as a safe and effective treatment option against hair loss even if more extensive controlled studies are needed to strengthen these results.

## Supplementary Materials

Supplementary materials can be found at https://www.mdpi.com/1422-0067/20/14/3446/s1.
