# Supplementary material for: Autologous Cellular Method Using Micrografts of Human Adipose Tissue Derived Follicle Stem Cells in Androgenic Alopecia
Source: Int J Mol Sci. 2019 Jul 13;20(14):3446. doi: 10.3390/ijms20143446 (PMC6678214; doi:10.3390/ijms20143446)
Supplement: Supplementary file 1 [file ijms-20-03446-s001.pdf]

Supplementary Table S1. Data analysis of hair count and hair density of 33 randomized patients treated with three micro-grafts injections before and 44weeks later the last treatment. Abbreviation: Patient (Pt); *Patient with trichoscan analysis reported in Figure 4A-D and with clinical outcome showed in Figure 8AB.

	**Total Hair Count - Baseline hairs x 0.65 cm^2**	**Total Hair Count - T5 hairs x 0.65 cm^2**	**Total Hair Density - Baseline hairs x cm^2**	**Total Hair Density - T5 hairs x cm^2**	**Odds Ratio (OR) (95% CI) *p*-Value**
Pt. 1 *	69,2	87,5	85,5	108,8	0.6539<br>(0.4275 to 1.0002)<br>$p = 0.05$
Pt. 2	67,4	85,5	81,3	109,1	0.6450<br>(0.4195 to 0.9919)<br>$p = 0.04$
Pt. 3	68,9	87,5	86,8	115,4	0.5982<br>(0.3919 to 0.9129)<br>$p = 0.01$
Pt. 4	72,9	92,5	91,2	121,2	0.6016<br>(0.3988 to 0.9076)<br>$p = 0.01$
Pt. 5	71,3	90,5	93,2	123,9	0.5586<br>(0.3702 to 0.8430)<br>$p = 0.005$
Pt. 6	74,8	94,9	95,1	131,2	0.5589<br>(0.3735 to 0.8365)<br>$p = 0.004$
Pt. 7	65,7	83,4	84,9	109,5	0.5892<br>(0.3826 to 0.9073)<br>$p = 0.01$
Pt. 8	81,7	103,7	100,2	131,2	0.6369<br>(0.4310 to 0.9411)<br>$p = 0.02$
Pt. 9	77,7	98,6	96,5	130,2	0.6046<br>(0.4060 to 0.9005)<br>$p = 0.01$
Pt. 10	91,7	114,6	112,4	149,4	0.6216<br>(0.4299 to 0.8989)<br>$p = 0.01$

Pt. 11	76,2	98,3	93,6	124,4	0.6459<br>(0.4318 to 0.9659)<br>*p* = 0.03
Pt. 12	87,4	106,6	106,5	141,6	0.6170<br>(0.4222 to 0.9016)<br>*p* = 0.01
Pt. 13	82,2	108,5	101,3	137,7	0.6400<br>(0.4354 to 0.9409)<br>*p* = 0.02
Pt. 14	90,2	117,2	110,1	143,1	0.6694<br>(0.4621 to 0.9697)<br>*p* = 0.03
Pt. 15	87,2	108,1	107,1	139,2	0.6317<br>(0.4327 to 0.9224)<br>*p* = 0.01
Pt. 16	75,8	96,2	96,7	131,5	0.5725<br>(0.3835 to 0.8547)<br>*p* = 0.006
Pt. 17	57,9	69,4	79,1	98,8	0.5080<br>(0.3209 to 0.8041)<br>*p* = 0.003
Pt. 18	65,3	87,5	86,7	122,2	0.5390<br>(0.3529 to 0.8232)<br>*p* = 0.004
Pt. 19	68,9	73,7	88,1	117,1	0.4821<br>(0.3134 to 0.7418)<br>*p* = 0.0009
Pt. 20	74,4	94,4	95,8	127,4	0.5765<br>(0.3848 to 0.8638)<br>*p* = 0.007
Pt. 21	65,7	88,6	85,4	113,5	0.5955<br>(0.3887 to 0.9123)<br>*p* = 0.01
Pt. 22	81,7	97,2	104,2	138,5	0.5474<br>(0.3708 to 0.8083)<br>*p* = 0.002
Pt. 23	77,1	97,9	98,2	130,6	0.5863<br>(0.3938 to 0.8727)<br>*p* = 0.008

Pt. 24	91,3	115,9	112,2	142,4	0.6580<br>(0.4545 to 0.9527)<br>*p* = 0.02
Pt. 25	76,2	99,8	87,9	122,1	0.7089<br>(0.4722 to 1.0641)<br>*p* = 0.09
Pt. 26	68,3	84,1	89,2	118,6	0.5439<br>(0.3567 to 0.8293)<br>*p* = 0.004
Pt. 27	72,9	92,5	93,4	124,2	0.5744<br>(0.3815 to 0.8648)<br>*p* = 0.007
Pt. 28	71,3	92,6	92,3	122,7	0.5820<br>(0.3857 to 0.8780)<br>*p* = 0.009
Pt. 29	63,6	78,8	85,7	113,1	0.5116<br>(0.3311 to 0.7906)<br>*p* = 0.002
Pt. 30	66,6	84,5	87,6	117,3	0.5447<br>(0.3559 to 0.8334)<br>*p* = 0.005
Pt. 31	70,5	89,5	92,3	122,7	0.5551<br>(0.3669 to 0.8396)<br>*p* = 0.005
Pt. 32	65,8	83,5	86,7	115,3	0.5455<br>(0.3554 to 0.8372)<br>*p* = 0.005
Pt. 33	76,6	81,9	98,9	131,5	0.4795<br>(0.3189 to 0.7211)<br>*p* = 0.0004
